# CKD273, a New Proteomics Classifier Assessing CKD and Its Prognosis

**DOI:** 10.1371/journal.pone.0062837

**Published:** 2013-05-14

**Authors:** Àngel Argilés, Justyna Siwy, Flore Duranton, Nathalie Gayrard, Mohammed Dakna, Ulrika Lundin, Lourdes Osaba, Christian Delles, Georges Mourad, Klaus M. Weinberger, Harald Mischak

**Affiliations:** 1 RD Néphrologie, Montpellier, France; 2 Néphrologie Dialyse St Guilhem, Sète, France; 3 Service de Néphrologie, Dialyse Péritonéale et Transplantation, Montpellier, France; 4 Mosaiques Diagnostics & Therapeutics AG, Hannover, Germany; 5 Charité-Universitätsmedizin Berlin, Berlin, Germany; 6 Biocrates life sciences AG, Innsbruck, Austria; 7 Progenika Biopharma, S.A., Derio, Vizcaya, Spain; 8 BHF Glasgow Cardiovascular Research Centre, University of Glasgow, Glasgow, United Kingdom; I2MC INSERM UMR U1048, France

## Abstract

National Kidney Foundation CKD staging has allowed uniformity in studies on CKD. However, early diagnosis and predicting progression to end stage renal disease are yet to be improved. Seventy six patients with different levels of CKD, including outpatients and dialysed patients were studied for transcriptome, metabolome and proteome description. High resolution urinary proteome analysis was blindly performed in the 53 non-anuric out of the 76 CKD patients. In addition to routine clinical parameters, CKD273, a urinary proteomics-based classifier and its peptides were quantified. The baseline values were analyzed with regard to the clinical parameters and the occurrence of death or renal death during follow-up (3.6 years) as the main outcome measurements. None of the patients with CKD273<0.55 required dialysis or died while all fifteen patients that reached an endpoint had a CKD273 score >0.55. Unsupervised clustering analysis of the CKD273 peptides separated the patients into two main groups differing in CKD associated parameters. Among the 273 biomarkers, peptides derived from serum proteins were relatively increased in patients with lower glomerular filtration rate, while collagen-derived peptides were relatively decreased (p<0.05; Spearman). CKD273 was different in the groups with different renal function (p<0.003). The CKD273 classifier separated CKD patients according to their renal function and informed on the likelihood of experiencing adverse outcome. Recently defined in a large population, CKD273 is the first proteomic-based classifier successfully tested for prognosis of CKD progression in an independent cohort.

## Introduction

Chronic kidney disease (CKD), by its high prevalence [1] and major impact on national care budgets [2], [3] is a concerning public health problem. The Global Burden Disease Study report has recently identified an increase in age standardized death rates from CKD, confirming these concerns [4] and measures are presently directed to early detection of CKD and prevention of its complications in order to improve survival and quality of life [5]. Among the causes of CKD, primary glomerular diseases have recently been overtaken by diabetes [2], which is increasingly observed even in young populations [6] and is associated with increased morbidity and mortality [7]. Despite different aetiologies, similar changes in renal architecture and function are observed with CKD progression. Still, the pathophysiology of renal disease is not completely elucidated and estimating the likelihood of disease progression is of utmost importance, as it influences follow-up and treatment.

In 2002, the National Kidney Foundation proposed a definition and classification of CKD, based on renal damage markers and serum creatinine-based estimation of glomerular filtration rate (eGFR) [8]. This classification has allowed unifying and standardizing CKD, thereby enabling better assessment of the natural history of CKD, independently of the aetiology, as well as to evaluate the efficacy of different treatments in improving outcomes. However, the classical renal function markers (serum creatinine level and GFR) are recognised to be late markers of disease, as organ damage forcedly precedes functional modifications such as filtration impairment. Single urinary markers, such as albumin, N-acetyl-glucosaminidase [9], α1-microglobulin [10], or neutrophil gelatinase-associated lipocalin [11] have been proposed as earlier markers. Microalbuminuria was demonstrated to be linked to developing overt CKD in diabetes [12]. Albuminuria is generally the consequence of glomerular dysfunction. Although increased albumin excretion may in turn increase renal damage, it is unlikely that albuminuria per se plays a significant role in the initiation of kidney disease. The identification of molecules linked to renal disease and likely to primarily participate in renal damage in CKD is eagerly sought. Such factors may be of help in assessing progression of disease, understanding pathophysiology, or, if participating early in the disease, in informing about the likelihood of kidney function loss [13].

Recently, significant progress has been achieved in the proteomic analysis of multiple compounds in biological fluids and high throughput analyses are now being applied, aiming at screening or diagnosing disease, unraveling pathophysiology, monitoring treatments, and establishing prognosis [14]. Proteomics allowed detection of differences in the urinary proteome between normo, micro and macroalbuminuric patients in type I diabetes and differentiated them from other chronic renal diseases15. Further, peptides identified in the urinary proteome have been combined to classifying models specific for different aetiologies of renal disease [15]–[19]. We have recently developed a classifier based on 273 urinary peptides (CKD273) which reliably allowed specific detection of CKD in a group of more than 600 patients and controls [20]. CKD273 classifier overcame the predominant patterns related to specific diseases, and identified CKD independently of the aetiology [20].

In the present study we investigated the classification performance of the proteomic classifier CKD273 [20] in a blind manner in a group of patients with different stages of CKD and assessed the association of the CKD273 classifier with the combined endpoint of loss of kidney function and death. The results validated the use of CKD273 classifier in assessing CKD and informing about outcome.

## Patients and Methods

### Patients

During three consecutive days, all patients eligible and attending the outpatient clinics of the hospitals of Sète and Montpellier, as well as the dialysis unit in Sète, were invited to participate in the study. Eligible were stable patients, over 18 year-old, not having been in hospital for over 2 months and not having acute inflammatory diseases. A total of 76 patients were included, of those, 53 were not anuric. Twenty seven of them were diabetics; 7 had nephroangiosclerosis, 4 interstitial nephritis, 3 chronic GN, 3 uninephrectomy, 3 autosomal dominant polycystic kidney disease, 3 other (anticalcineurine toxicity, diuretic abuse and sarcoidosis) and 3 unknown. For evaluation, renal function was estimated by simplified MDRD formula [21]. Their characteristics are given in Table 1 and the demographic, clinical and routine chemistry data of each of the 53 patients are given in Table S1.

**Table 1:** Patients’ characteristics.

N	53
M/F	33/20
D/ND	27/26
Age (years)	70±1.6
Serum creatinine (µmol/L)	249±23
CRP (mg/L)	4.22±0.51
WCC (cell/mm^3^)	6698±272
Hemoglobin (mg/dL)	13.1±0.2
Glycated Hemoglobin (%)	6.3±0.2
Serum proteins (g/L)	72.5±0.9
Serum calcium (mmol/L)	2.28±0.02
Serum phosphate (mmol/L)	1.19±0.03
Alkaline phosphatase (UI/L)	71.4±4.2
iPTH (pmol/L)	18.75±3.2
Urine proteins (g/g of creatinine)	1.18±0.32
eGFR (mL/min/1.73 m^2^)*	36.01±2.98

Abbreviations: CRP (C reactive protein); WCC (white cell counts), iPTH (intact parathyroid hormone); eGFR (estimated glomerular filtration rate).

For continuous variables, results are presented as mean ±SEM.

* 6 patients were treated with haemodialysis. The eGFR and urinary concentration of creatinine do not apply to these patients given that creatinine levels vary following dialysis treatment.

The study was designed and conducted fulfilling all the requisites of the French law on the protection of individuals collaborating in medical research and was in accordance with the principles of the Declaration of Helsinki. Written informed consent was obtained from all participants. The data were handled according to the rules of the CNIL (Centre National d’Informatique et Liberté) warranting the respect of privacy. It was declared to the French Ministry with the allocated reference number DC – 2008 – 417 and was approved by the local ethics committee, the Comité de Protection de Personnes (CPP) of Montpellier.

All patients underwent clinical examination and had routine laboratory screening including the determination of creatinine, urea, glucose, calcium and phosphate serum levels as well as blood cell counts, during morning. Fasting was not demanded. Fresh urine was collected and checked for routine chemistries. Two aliquots were frozen immediately for proteomics analyses as described below, and stored at −80°C until analysis.

The samples were coded and shipped to the laboratory for urinary proteomics analysis, which was performed blinded, without any further information. The samples were unblinded after depositing the results by the proteomics laboratory (Mosaiques).

The patients were subsequently seen regularly in the outpatient clinic and clinical and laboratory data recorded. Physicians were not informed of CKD273 classifier scoring. Patient management during the follow-up period was only based on normal practice. When patients did not attend the clinic, data were obtained from general practitioners. Deaths or initiation of renal replacement therapy were assessed as a combined endpoint. After 3.6 years of follow-up, outcome was obtained from 49 patients (4 patients were lost-to-follow up, 7.5%). Fifteen patients reached an endpoint. Of those, nine patients started dialysis and six patients died not being on dialysis. Thirty-three patients did not reach an endpoint. One patient was transplanted and was not included in the follow-up analyses.

## Methods

### Sample preparation

Samples were prepared as described [20]: a 0.7 mL aliquot stored urine was thawed and diluted with 0.7 mL 2 M urea, 10 mM NH_4_OH containing 0.02% SDS. Samples were filtered using Centrisart ultracentrifugation filter devices (20 kDa cut-off; Sartorius, Goettingen, Germany) at 3,000 g until 1.1 mL of filtrate was obtained. Subsequently, filtrate was desalted using PD-10 column (GE Healthcare, Sweden) equilibrated in 0.01% NH_4_OH in HPLC-grade water. Finally, samples were lyophilized and stored at 4°C. Shortly before CE-MS analysis, lyophilisates were re-suspended in HPLC-grade water to a final protein concentration of 0.8 µg/µL checked by BCA assay (Interchim, Montlucon, France).

### CE-MS analysis

Capillary electrophoresis–coupled mass spectrometry (CE-MS) analysis was performed as described [15], [22], [23]. The average recovery of sample in the preparation procedure was ∼85% and the limit of detection was ∼1 fmol. Mass resolution was above 8,000 Da enabling resolution of monoisotopic mass signals for z≤6. After charge deconvolution, mass accuracy was <25 ppm for monoisotopic resolution and <100 ppm for unresolved peaks (z>6). The analytical precision of the platform was assessed extensively [15], [20].

### Proteomics data processing and evaluation

Mass spectral peaks representing identical molecules at different charge states were deconvoluted into single masses using MosaiquesVisu software [24]. Only signals with z>1 observed in a minimum of 3 consecutive spectra with a signal-to-noise ratio of at least 4 were considered. Reference signals of 1770 urinary polypeptides were used for CE-time calibration by locally weighted regression. For normalization of analytical and urine dilution variances, signal intensities were normalized relative to 29 “housekeeping” peptides [25]. The obtained peak lists characterize each polypeptide by its molecular mass [Da], normalized CE migration time [min] and normalized signal intensity. All detected peptides were deposited, matched, and annotated in a Microsoft SQL database allowing further statistical analysis. For clustering, peptides in different samples were considered identical if mass deviation was <50 ppm. CE migration time was controlled to be below 0.35 minutes after calibration.

### Statistical analysis

The analyses of proteomic data were performed in a blind manner and the results (amplitude of single peptides and scoring of the CKD273 classifier) were stored in the central data base before unblinding. Then the codes were opened and the data were merged for the unblinded analysis. The scorings of the CKD273 classifier were tested by general linear model (GLM) analysis for unbalanced data. For clustering analysis, patients were assigned to clusters based on their respective 273 proteome pattern following an average linkage clustering using standard Euclidean distances. The two main clusters identified were analyzed. Cluster membership was taken as a two-level class variable and parametric and non parametric tests were applied to assess differences in clinical variables. Analyses were performed using SAS version 9.1 (SAS inc, North Carolina, USA). The results are given as mean ± SEM.

## Results

**Figure 1:**
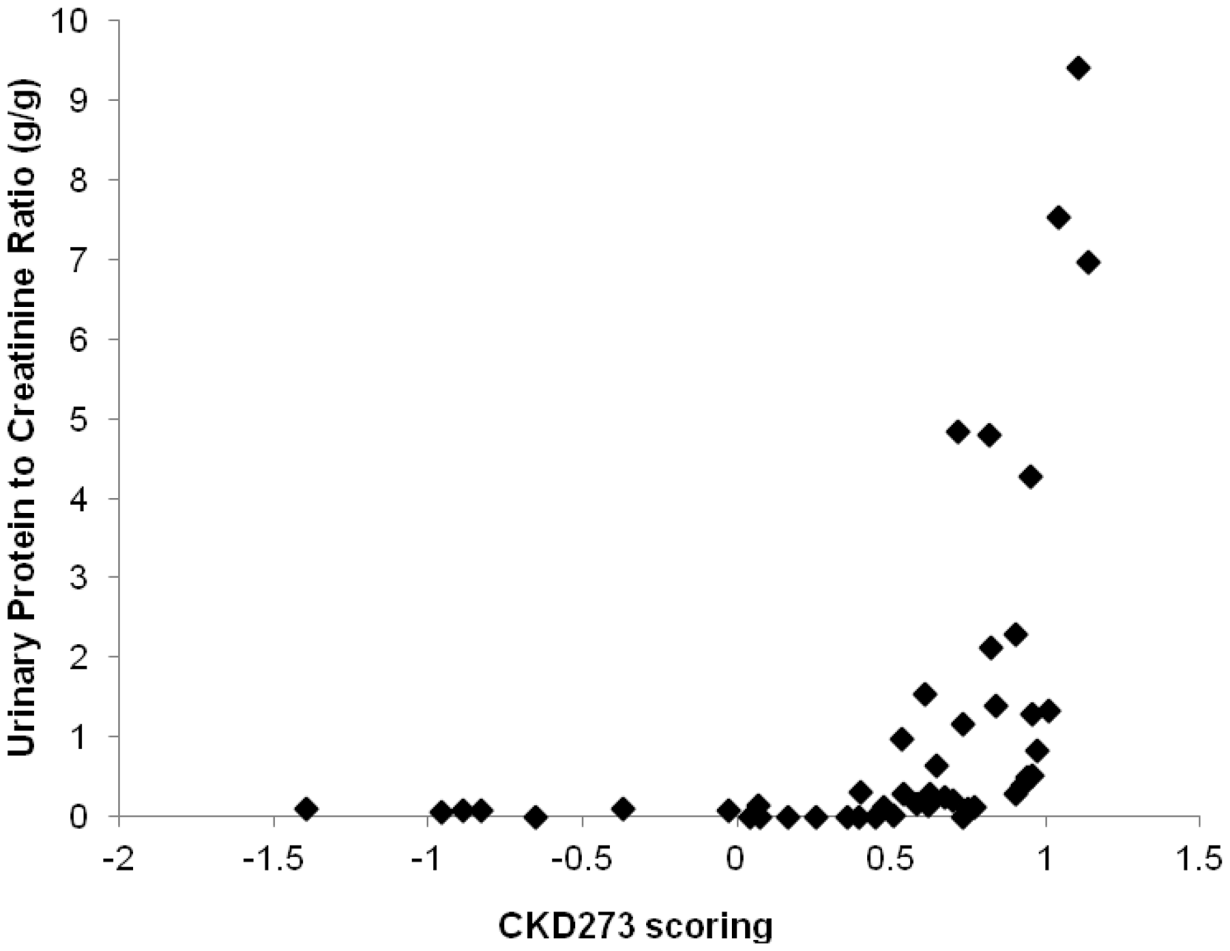
The full urinary proteome/peptidome analysed by CE-MS of selected patients. Patient number 58 (A) and the diabetic patient 27 (B) are shown in the upper panels. These patients had eGFR of 64.3 and 15.7 ml/mn/1.73 m^2^, respectively. The molecular mass (0.8–20 kDa, on a logarithmic scale) is plotted against normalized migration time (18–45 min). Signal intensity is encoded by peak height and color. The two lower panels show only the peptides that are addressed in the CKD273 model. Differences already visible in the entire proteome become evident when examining the specific biomarkers of the CKD273 classifier.

The demographic, clinical and routine chemistry data of each of the 53 patients are given in Table 1 and Table S1. Urine samples collected in the outpatient clinic were blindly analyzed employing the established CE-MS proteomics platform. The resulting data were scored with the previously established CKD273 classifier, which combines 273 distinct urinary proteomic biomarkers associated with CKD into one numeric variable. Figure 1 presents illustrative examples of urinary proteomes of two included patients at different stages of CKD.

**Figure 2:**
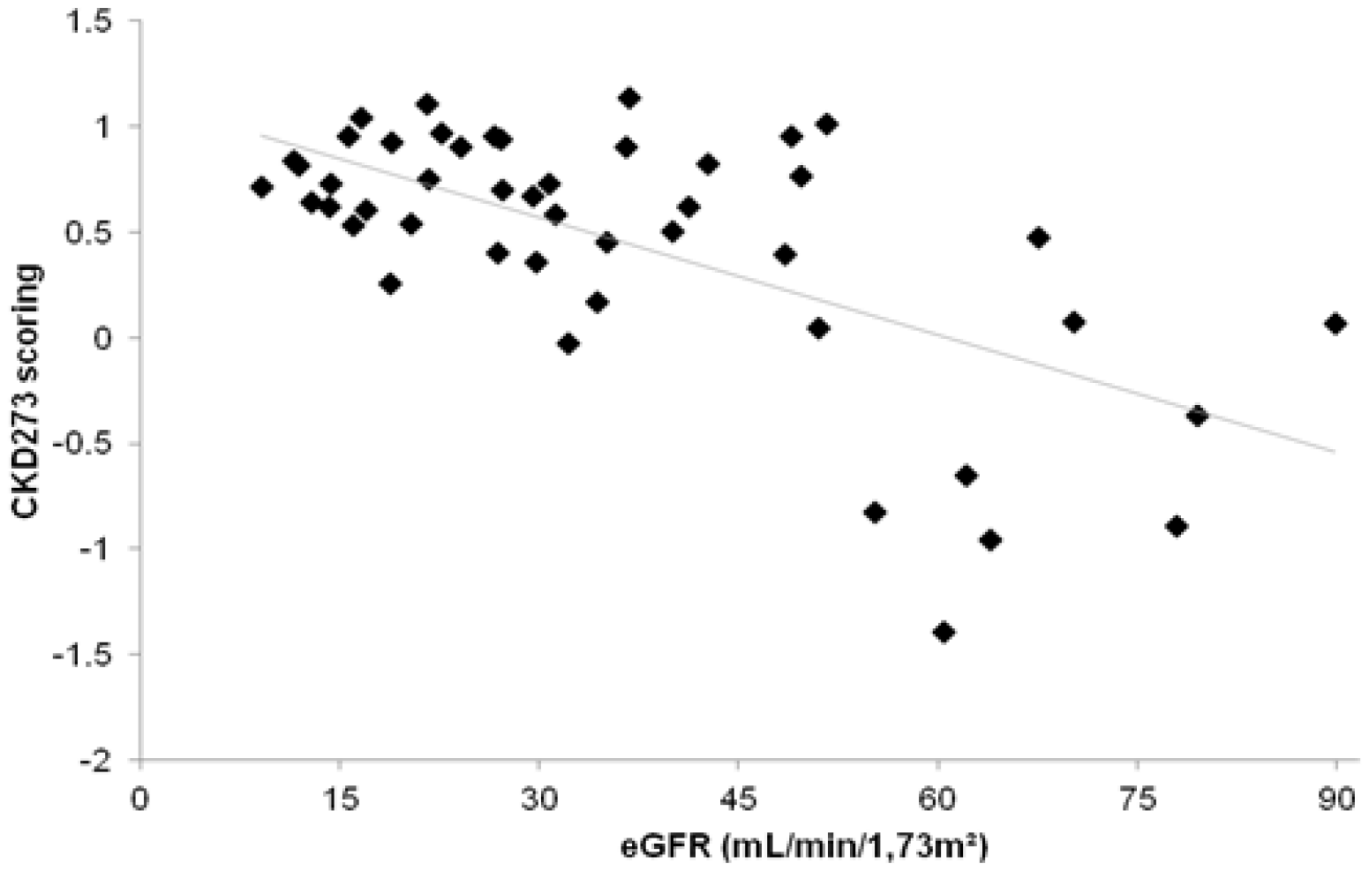
CKD273 scoring and eGFR values in the patients included in the study. The patients treated by dialysis are not included in the figure as their eGFR could not be calculated by the serum creatinine level. Linear Regression equation, y = −0.019x +1.12; R^2^ = 0.40; p<0.001.

General linear model (GLM) analyses of the CKD273 classifier showed a significant difference in the patients distributed according to their eGFR into 4 different groups following the National Kidney Foundation classification (stages I-II, III, IV and V CKD) (F = 19.44; p<0.0001). CKD273 score was linearly correlated with eGFR (R = −0.64; p<0.001) (Figure 2), and with urinary protein excretion (g/g), in a non-linear way (ρ = 0.76; p<0.001) (Figure S1).

**Figure 3:**
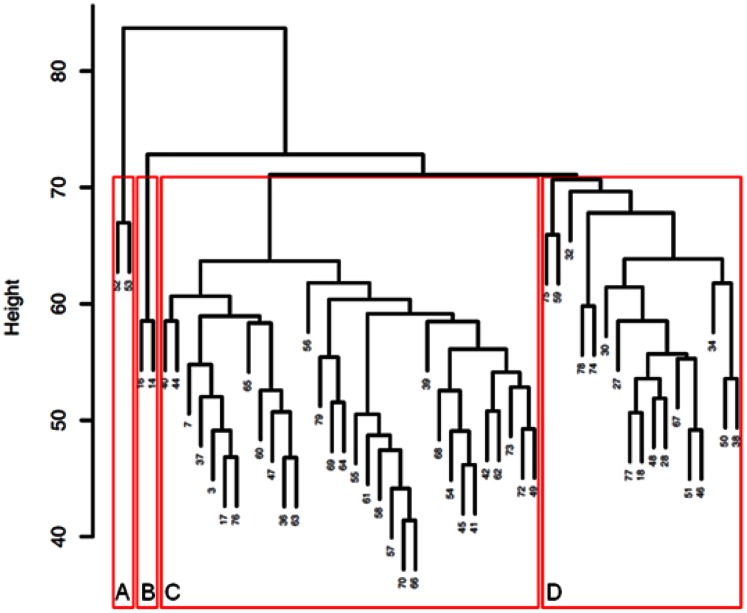
Cluster analysis. Clustering of the 53 patients was performed according to their CKD273 pattern using an average linkage clustering and standard Euclidean distances to assign the patients to clusters.

Unsupervised clustering analysis taking into account the individual values of the 273 peptides of the classifier separated the patients into two small clusters (A and B, 2 patients each) and two main clusters (C and D, 32 and 17 patients respectively) (Figure 3). Cluster A included 2 diabetic patients in the 70 s with heavy proteinuria and cluster B consisted of 2 diabetic patients with important cardio-vascular co-morbidities (both died from cardiac and vascular complications). There were significant differences in laboratory parameters between cluster C and D. Cluster C had a lower CKD273 scoring (p<0.0001) and higher eGFR (p<0.01). Patients in cluster C also had lower blood urea (p = 0.014), uric acid (p<0.01), urinary proteins (p = 0.002), PTH (p = 0.01) and alkaline phosphatase levels (p<0.002) and higher serum calcium level (p = 0.04), and red blood cell count (p = 0.013) (Table S2).

To obtain further insight into the pathophysiological changes associated with CKD, we investigated the correlation of the single peptides that comprise the CKD273 pattern, with the estimated glomerular filtration rate (eGFR). Several peptides revealed highly significant correlations with the eGFR (Table S3).

Among the peptides that showed an inverse correlation with eGFR, – increasing urinary relative concentrations with decreasing renal function – most were derived from abundant plasma proteins: β2-microglobulin, albumin, transthyretin, α-2HS glycoprotein, APO A1 and α1-antitrypsin. In contrast, the peptides with a positive correlation with eGFR – decreasing relative urinary concentrations with progressive impairing in renal function – were predominantly derived from constitutive renal proteins and extracellular matrix : collagen type I (29 peptides) and type III (8 peptides), Uromodulin, a protein locally synthesized in the renal tubule (5 peptides), osteopontin and CD99.

**Figure 4:**
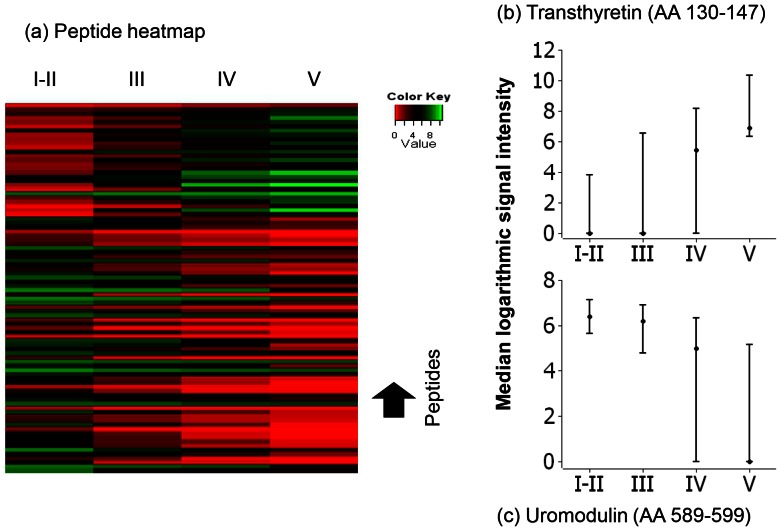
Correlation of urinary peptides with kidney function. a) Heatmap of the signal of the 88 peptides of the CKD273 model showing monotone variations. Each line represents a single peptide, and the four columns correspond to the four groups of patients separated according to their eGFR value (90–61, 60–31, 30–16 and 15–0 mL/min/1.73 m^2^). The mean signal per peptide and per group is represented following the color key included in the figure. **b)** and **c)**: the median scoring and interquartile range for 2 of the 88 peptides is presented in logarithmic scale. The upper panel shows the distribution of LLSPYSYSTTAVVTNPKE, a peptide derived from Transthyretin (AA 130–147). The lower panel depicts the distribution of SGSVIDQSRVL, a peptide derived from Uromodulin (AA 589–599). The abundance of these peptides changes significantly with decreasing MDRD-estimated GFR.

A heatmap of the mean signal intensity of those peptides of the CKD273 classifier which were present in >50% of the samples and showed monotone variations, averaged in the four different groups (stage I-II, III, IV and V) revealed a clearly distinct pattern for the four groups (Figure 4-a). The median signal intensity for 2 of these peptides is plotted to illustrate the magnitude of the differences observed between the groups according to their estimated renal function (Figure 4-b and 4-c).

**Figure 5:**
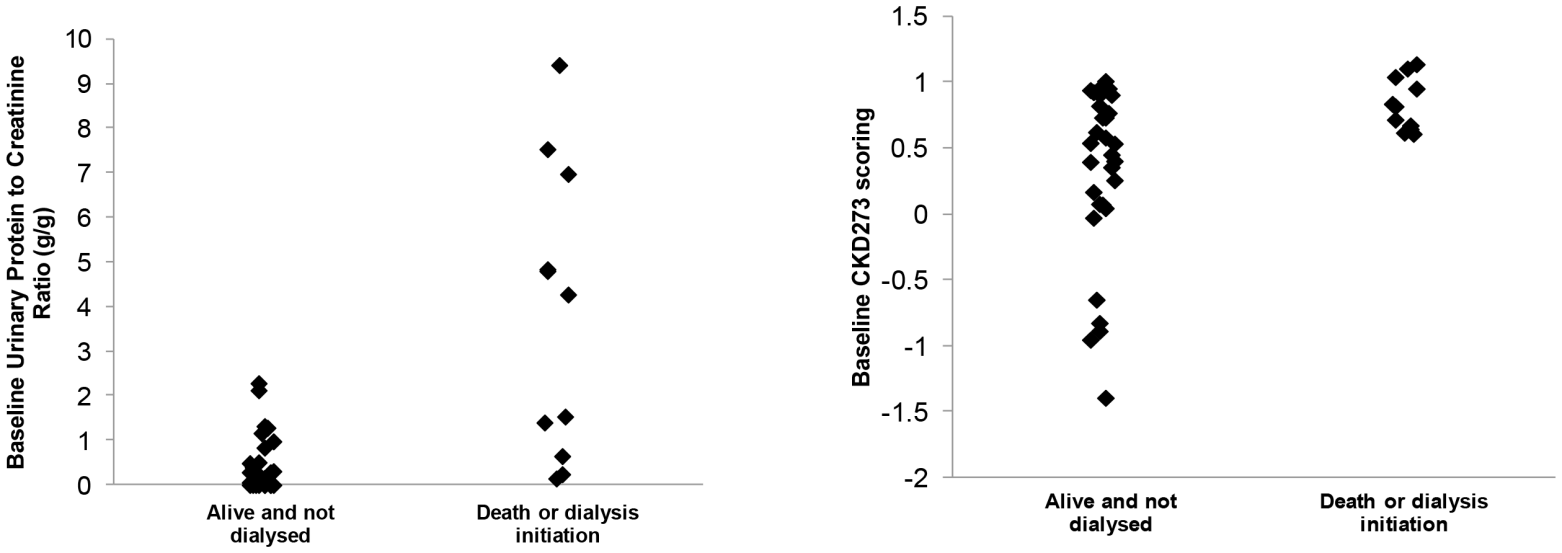
Urinary protein excretion and CKD273 scoring at baseline and renal outcome. Patients were divided into 2 groups according to their evolution during the follow-up period: Those that stayed alive and did not need dialysis and those that either died or needed dialysis. It can be observed that urinary protein excretion was distributed in a wide range in the group of patients that needed dialysis or died (from normal to nephrotic range), showing that although a high urinary protein excretion invariably resulted in renal failure progression, a low urinary protein excretion did not preclude death or dialysis. At the right hand side can be observed that the CKD273 scoring of the patients that either died or needed dialysis was clearly in a narrower range. None of the patients with low scores of CKD273 (<0,55) progressed to renal failure or died; a low CKD273 score precluded death or need of dialysis in this population followed up for 3.6 years.

After 3.6 years of follow-up, the outcome was known for 49 patients (92.5%). All the patients having lost their renal function or having died during follow-up had a very high CKD273 score (Figure 5). There was a clear CDK273 cut-off value below which patients did not progress to death or renal death during the follow-up period. Conversely, urinary protein excretion showed good specificity in identifying patients with high likelihood of end-stage renal disease or death.

CKD273 score was correlated in a non-linear way with urinary protein excretion expressed as g of protein/g of creatinine (ρ = 0.76; p<0.001) (shown in Figure S1). Despite the existing relationship between CKD273 and urinary protein excretion (Figure S1), their association with outcome was substantially different (Figure 5). While a low score in the CKD273 classifier identified those patients that would certainly not evolve to renal loss or death, high urinary protein excretion would identify those that would certainly evolve to renal loss and death during the follow-up period (Figure 5).

## Discussion

CKD273, a urinary proteomics based classifier which was developed to detect CKD irrespective of the underlying aetiology [20], was able in the present study to blindly discriminate CKD patients according to disease severity. Confirming the validity of the data, unsupervised clustering analysis identified two main groups that had different levels of renal function as demonstrated by significant differences in blood urea level, eGFR, haemoglobin and mineral bone markers status. The CKD273 classifier was associated with the renal function level cross-sectionally at the beginning of the follow-up study. More importantly, CKD273 score was significantly higher in the patients who evolved towards end-stage renal disease or died during the following years.

The information provided by the CKD273 classifier may be exploited in two different ways. The first one is the intended value of the classifier obtained as a composite single value in assessing CKD, as supported by our present results. The second is to analyze the different constitutive peptides separately following their respective readouts. Individual analysis of the constitutive peptides of the CKD273 classifier showed clear differences in urinary levels of specific compounds that correlate with the degree of renal function impairment as measured by eGFR. Since all 273 peptides constitutive of the CKD273 classifier are known, the detection and quantification of these various compounds may offer novel insights as markers of disease or perhaps even as compounds involved in the disease process, although the latter has not yet been proven.

The majority of peptides positively correlated with eGFR was derived from collagen type I and type III, and from the locally synthesized Uromodulin. The decrease in the abundance of these peptides in urine may be related to a decrease in extracellular matrix degradation, turnover, and organ repair, as well as to fibrosis, which has been identified as a characteristic feature of the late stages of renal diseases [26]. The data presented here argue for a significant involvement in earlier stages of disease as well. These findings are consistent with a previous report in diabetic patients [17], showing that a hallmark of diabetic nephropathy is a reduction in urinary collagen fragments. They are also in keeping with Prajczer *et al* [27], who observed very low levels of urinary Uromodulin as eGFR decreases. Osteopontin, a glycoprotein highly expressed in renal failure [28], may also be related to fibrosis, as it facilitates calcium oxalate monohydrate development of interstitial fibrosis in hyperoxaluric states [29], [30]. Based on our results, these peptides and proteins likely display different facets of the molecular pathophysiology of this heterogeneous disease.

Beyond the interest in identifying peptide products of different pathways, clinical proteomics is primarily orientated at complementing and improving the classical biomarkers, such as serum creatinine level or albumin/creatinine urinary ratio. The CKD273 classifier previously proved to dissociate cases from controls in a population including 230 patients with biopsy proven kidney disease and 379 healthy subjects [20]. In the present work, we show its correlation with CKD stage irrespective of the causal kidney disease.

The longitudinal analyses of the clinical and biochemical data support that CKD273 may inform about the likelihood of eGFR decline and predicting CKD progression to end stage kidney disease. The progress in proteomics and the use of multiple biomarker classifiers open the possibility of establishing new tools adapted to different clinical needs. Some of them have been built up to differentiate aetiology, others to assess CKD and CKD progression. It is expected that with a more common use of proteomics and with the sequence identification of the complete urinary proteome, additional classifiers will be developed for specific clinical applications, e.g. assessing specific CKD aetiology like DN, and predicting both, disease progression and response to therapy, based on prespecified molecular features.

Our present data show that proteomics may provide supplementary information on aspects of CKD mainly unrelated to serum creatinine level and derived estimates of glomerular filtration rate, which inform about the likelihood of disease progression. Indeed, National Kidney Foundation–CKD staging has also allowed testing of the contribution of these technologies in handling CKD patients. Although not routinely available, proteomics is becoming more and more accessible, with increasing automation easing application and reducing costs. Proteomics has enabled identification of new biomarkers of CKD with promising clinical value [20]. The present study provides the first set of data validating the CKD273 classifier in an independent cohort, making it ready to be investigated for its prognostic value in large populations on a long-term longitudinal basis.

## Supporting Information

Figure S1 Correlation studies of Urinary protein excretion and CKD273 classifier scoring. It can be observed that urinary protein excretion and CKD273 scoring followed an exponential function and were significantly correlated (Spearman ς = 0.76; p<0.0001).
(TIF)

Table S1 Clinical data and CKD273 classifier scoring of the individual subjects in the study.
(XLS)

Table S2 Variables with significant differences when comparing the 2 main clusters identified with the values of the CKD273 peptides (clusters C and D).
(XLS)

Table S3 Urinary peptides of the CKD273 model, which significantly correlated with eGFR.
(XLS)
